# Uses of Local Plant Biodiversity among the Tribal Communities of Pangi Valley of District Chamba in Cold Desert Himalaya, India

**DOI:** 10.1155/2014/753289

**Published:** 2014-02-17

**Authors:** Pawan Kumar Rana, Puneet Kumar, Vijay Kumar Singhal, Jai Chand Rana

**Affiliations:** ^1^Department of Botany, Punjabi University, Patiala, Punjab 147002, India; ^2^National Bureau of Plant Genetic Resources, Regional Station, Phagli, Shimla 171003, India

## Abstract

Pangi Valley is the interior most tribal area in Himachal Pradesh of Northwest Himalaya. An ethnobotanical investigation is attempted to highlight the traditional knowledge of medicinal plants being used by the tribes of Pangi Valley. Various localities visited in the valley 2-3 times in a year and ethnobotanical information was collected through interviews with elderly people, women, shepherds, and local vaids during May 2009 to September 2013. This paper documented 67 plant species from 59 genera and 36 families along with their botanical name, local name, family name, habit, medicinal parts used, and traditional usage, including the use of 35 plants with new ethnomedicinal and other use from the study area for the first time. Wild plants represent an important part of their medicinal, dietary, handicraft, fuel wood, veterinary, and fodder components. These tribal inhabitants and migrants depend on the wild plant resources for food, medicines, fuel, fibre, timber, and household articles for their livelihood security. The present study documents and contributes significant ethnobotanical information from the remote high altitude and difficult region of the world, which remains cut off from rest of the world for 6-7 months due to heavy snowfall.

## 1. Introduction

The cold arid region of India also called “Trans Himalayan region” lies in the western edge of the Himalayas. It comprises Ladakh in J&K, Lahaul and Spiti, Kinnaur, Pangi Valley of district Chamba in Himachal Pradesh, and Niti and Nelong Valley of Uttarakhand. The vegetation here is subjected to extreme climatic conditions such as temperature variation (low temperature), scanty rainfall, speedy winds, exposure to ultraviolet radiations, reduced oxygen levels, low humidity, and many small glaciers. Pangi Valley, a subdivision of Chamba district is the remote high-altitudinal area and one of the most beautiful valley in the Northwest Himalaya. The river Chandrabhaga flows through deep narrow gorges in the Pangi Valley. It originates from Baralacha glacier in Lahaul-Spiti and enters in Pangi Valley near Karhu Nala. It becomes the Chenab when it joins the Marau River at Bhandera Kot, 12 km from Kishtwar town in Jammu and Kashmir. The Valley has recently been connected by road via Sach Pass at a height of 4,350 m, the highest road in Himachal Pradesh. It is also the shortest route from Chamba to Killar (170 km) and is open for vehicular traffic between mid June and September, but it remained closed due to heavy snowfall at other times of the year. One can approach the Valley via Chamba-Manali-Killar (680 km) and Chamba-Jammu-Doda-Gulabgarh-Killar (570 km), but these are very long routes compared to the Chamba-Sach Pass-Killar route. The old trade routes still exist which connect Pangi Valley to Ladakh of Zanskar range in the adjoining state of Jammu and Kashmir. Practically all the people live in small and fairly isolated villages. The languages spoken by the people are *Pangwali* and *Bhoti*. Both Hinduism and Budhism are practised in the valley. The tribal people of Pangi are called the “*Pangwal*.” The high altitudinal villages of Pangi Valley are called *Bhatories* and their residents are referred to as “*bhots*.” These people are mostly Buddhists and have Tibet-Mongolian features. Adjoining hills of Pangi Valley towards the southern side are visited frequently by migratory pastoralist tribal *Gaddis* and nomadic *Gujjars* with their herds. These migrants go to higher altitudes in summer along with their herds in search of grazing ground and meadows. They also collect different parts of various medicinal and aromatic plants for their earnings. Local songs, dance (*Nati* by gents and *Ghurei* by ladies), and locally brewed liquor “*paatar*,” play a significant role on the life style of the people of Pangi Valley. One of the major festivals celebrated in the mid of February is “*Jukaru*,” praying to local god or deities with words of celebration and thanks for helping the people to survive the harsh winter. One is compelled to think of how and why people thought of settling down in this most inaccessible part of the state. The vegetation of Pangi can be broadly categorized into three types:- Himalayan Temperate Forests—At low altitude between 1,900–2,800 m; the vegetation is typically of *Himalayan temperate type*. *Pinus gerardiana, Cedrus deodara, Pinus wallichiana, Picea smithiana*, *Abies spectabilis, Taxus baccata* ssp. *wallichiana, Juniperus macropoda, Populus ciliata, Salix viminalis, Crataegus songarica, *and* Acer pentapomicum *forming the top canopy. The second storey is constituted by* Fraxinus xanthoxyloides*, *Rhus succedana, Parrotiopsis jacquemontiana*, and *Olea ferruginea. *Shrubby and scrub elements include species of *Berberis lycium, Ribes orientale, R. nigrum, Rosa webbiana, Viburnum cotonifolium, Lonicera quinquelocularis, Hippophae rhamnoides, Myricaria squamosa, Daphne oleoides, Rubus saxatilis*, and *Sorbus foliosa. Rabdosia rugosa*, *Ephedra gerardiana, Artemisia brevifolia, A. maritima, *and* A. parviflora *form dense scrubs covering vast tracts of slopes in the region. At altitude between 2,800–3,800 m, the vegetation is *subalpine type* represented by *Allium humile, Bunium persicum, Carum carvi, Geranium wallichianum, Angelica glauca, Bupleurum falcatum, Elsholtzia ciliata, Heracleum lanatum, Arisaema flavum, Primula denticulata, P. macrophylla, Saussurea costus, S. auriculata, Tanacetum gracile, T. tomentosum, T. tenuifolium, Impatiens glandulifera, Arnebia benthamii, Eritrichium canum, Ranunculus laetus, R. hirtellus, Rhododendron campanulatum, Ribes orientale, R. alpestre, Polygonatum multiflorum, P. verticillatum, Plantago depressa, Lepidium latifolium, Polygonum sibiricum, Potentilla atrosanguinea, Anemone obtusifolia, Aconitum ferox, A. falconeri, Dactylorhiza hatagirea, Picrorhiza kurroa, Pedicularis pectinata, Elymus dahuricus, Aesculus indica, Corylus jacquemontii, *and *Juglans regia. Alpine zone* ranging from 3,800 m onwards, the vegetation is mainly dominated by species of *Betula utilis, Rhododendron campanulatum, Myricaria squamosa, Capparis himalayensis, Cassiope fastigiata*, *Cortia depressa, Selinium tenuifolium, Heracleum wallichii, Inula royleana, Saussurea graminifolia, S. obvallata, S. gossypiphora, Arnebia euchroma, Corydalis meifolia, Iris kumaonensis, Fritillaria roylei, Polygonum affine, Rhododendron anthopogon, Rheum spiciforme, R. moorcroftianum, Rhodiola imbricata, Rheum australe, Picrorhiza kurroa, Aconitum heterophyllum, A. rotundifolium, A. violaceum, A. spicatum, Elymus nutans, E. dahuricus, Delphinium cashmerianum, D. vestitum, *and* Nardostachys grandiflora. *Other herbaceous vegetation consists of *Saussurea jacea, Triglochin maritima, Aquilegia fragrans, Potentilla* spp., *Onosma hispidum, Spinosa stracheyi, Geranium wallichianum, Jurinea macrocephela, Picrorhiza kurroa, Dracocephalum heterophyllum, Impatiens brachycentra*, and *Primula macrophylla*. Covering an area of 103 sq km, Saichu Tuan Nala Wild Life Sanctuary has been established in the valley for the protection and conservation of wildlife. The wild animals found in the valley are ibex, himalayan tahr, brown bear, black bear, musk deer, snow leopard, and bharal. The birds include the monal and koklas pheasants, himalayan western tragopan, snow peacock, snow pigeon, and chukor. This area was difficult to explore due to its remoteness, difficult geographic condition, and poor connectivity via roads due to heavy snow fall during winter season which keeps the area cut off from rest of the world for nearly six to seven months. These tribal inhabitants and migrants are dependent on the wild plant resources for medicines, food, fuel, fibre, timber, and household articles to a great extent for their livelihood security. The area due to its remoteness and difficult geographic conditions has not been included in the earlier floristic surveys of Chamba district by Singh and Sharma [1]. So far very few workers have visited the area for taxonomical studies [2] and for some cytological studies of dicot plants [3–9]. No information on traditional use of plant resources of Pangi Valley and its adjoining areas is available so far. Keeping in view the nonavailability of ethnobotanical information, strong belief of local people in traditional therapy, and scope for inventorization of new medicinal and common use, the present study was designed to provide comprehensive information on traditional phytotherapy and ethnobotanical information in cold desert region of Pangi Valley in Northwest Himalaya.

## 2. Materials and Methods 

**Figure 1:**
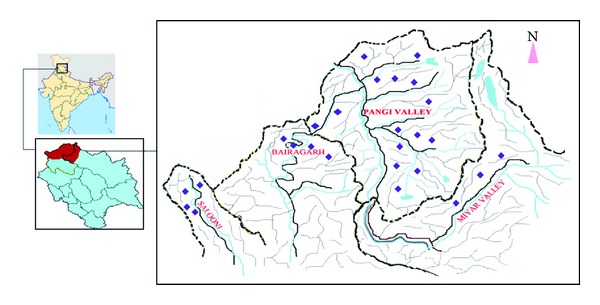
Location map of Pangi Valley in District Chamba (H.P.), Northwest Himalaya showing visited localities.

Ethnobotanical surveys were carried out from May 2009 to September 2012, when the area is snow-free. Different localities visited in the Pangi valley 2-3 times a year to document the utilization of medicinal plants (Figure 1) including Tarela (1,850 m), Bairagarh (1,900 m), Salooni (1,950 m), Dind (2,100 m), Bhandal (2,200 m), Devi Kothi (2,400 m), Shour (2,400 m), Hillour (2,450 m), Mindhal (2,500 m), Muhani (2,600 m), Sahali (2,600 m), Saichu-Nala (2,650 m), Killar (2,650 m), Chask (3,150 m), Twan (3,300 m), Satrundi (3,300 m), Kala Ban (3,350 m), Hillour Dhar (3,350 m), Udeen (3,400 m), Sural-Bhatori (3,400 m), Kumar-Bhatori (3,400 m), Chask-Bhatori (3,600 m), Bagotu (4,100 m), Singh-Marh Dhar (4,300 m) and Sach Pass (4,350 m), and Shakoli (3,200 m), Shitikar (3,650 m), and Urgos (3,800 m) in Miyar Valley. First-hand information on traditional knowledge related to plant resource utilization by the inhabitants of the valley is gathered through interviews with elderly people, women, shepherds, and local vaids. Information about the local names of the plants, parts used, ailments treated, mode of administration, and curative properties was recorded. The plants were identified by Flora of Lahaul-Spiti [10] and Flora of Chamba District [1]. Besides, the plants were also compared to the samples preserved in the Herbarium (PUN) (PUN is the Herbarium Code of Department of Botany, Punjabi University, Patiala as per “Index Herbariorum” by Holmgren and Holmgren, (1998), maintained by the Department of Botany, Punjabi University, Patiala and also the Herbaria of Botanical Survey of India and Forest Research Institute, Dehra Dun, Uttarakhand. Voucher specimens of the ethnobotanically studied species were deposited in the Herbarium, Department of Botany, Punjabi University, Patiala (PUN). Plants are enumerated in alphabetical order followed by accession number, habit, family, local names, parts used, and mode of preparation.

## 3. Results

**Figure 2:**
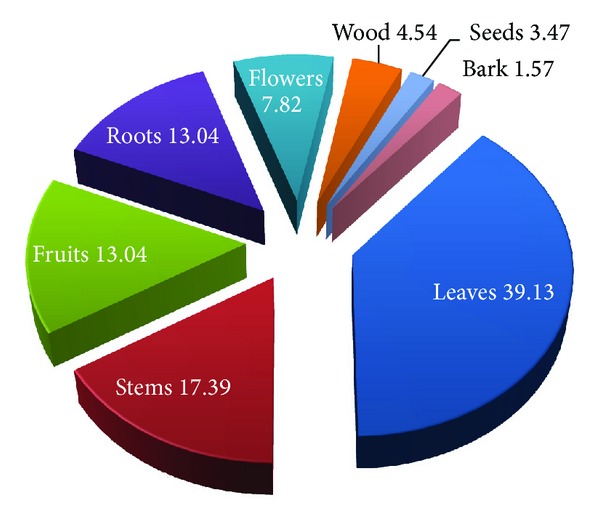
Percentage of plant parts used for medicinal and other important uses.

**Figure 3:**
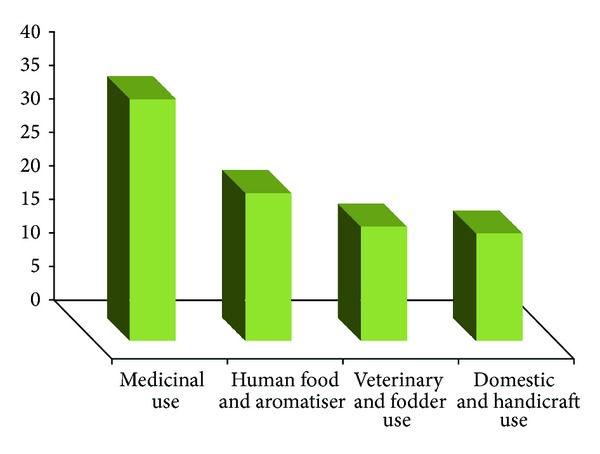
Four main categories of plants use in study.

**Figure 4:**
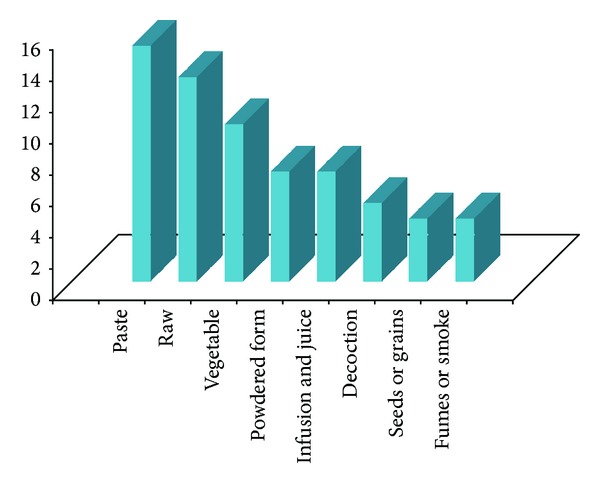
Mode of utilization of plants.

**Figure 5:**
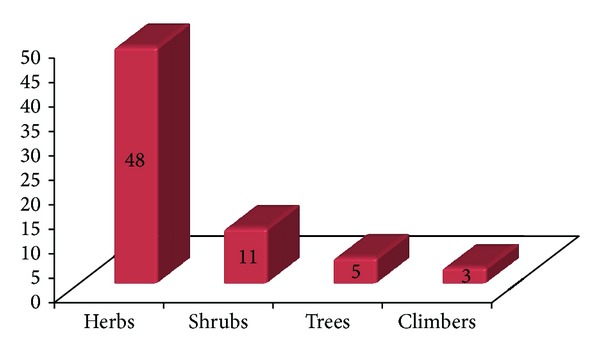
Life form of reported common plants.

This paper documented for the first time traditional uses of 67 plant species from 59 genera and 36 families along with their botanical name, local name, family, habit, plant part used, and local usage of application, from the remote, interior, and tribal area of Pangi Valley and its adjoining areas of district Chamba from cold desert region of Northwest Himalaya. All the plants are studied ethnobotanically for the first time from the tribal area. Plants belong to 36 families, of which the Asteraceae are represented by seven species. Papilionaceae, Ranunculaceae, Rosaceae, and Polygonaceae are represented by five species each. Caprifoliaceae and Lamiaceae are represented by three species and Apiaceae, Berberidaceae, Caryophyllaceae, Chenopodiaceae, Morinaceae, and Scrophulariaceae by two species each. Araliaceae, Balsaminaceae, Boraginaceae, Buxaceae, Cuscutaceae, Datiscaceae, Elaeagnaceae, Gentianaceae, Loranthaceae, Malvaceae, Moraceae, Oleaceae, Phytolaccaceae, Rubiaceae, Salicaceae, Saxifragaceae, Smilacaceae, Solanaceae, Ulmaceae, Urticaceae, Valerianaceae, and Violaceae are represented by one species. It is very important to underline that great majority of the plants grow wild. Only two wild plants *Inula racemosa* and *Saussurea costus* are domesticated and cultivated for medicinal or commercial purposes. However, people also trying to grow some medicinal plants such as *Aconitum heterophyllum, Podophyllum hexandrum, Angelica glauca, Valeriana jatamansi, *and* Picrorhiza kurroa *as kitchen garden plants at high altitudinal villages of Sural-Bhatroi, Hudan-Bhatorri, and Devi Kothi for their use and marketing purpose at local level. Different parts of the plants in powdered form/plant extracts/decoctions/concoction or paste are administered in various human ailments and other uses in their daily life. In most of the cases leaves are used followed by stems, fruits, roots, and flowers. Wood, seeds, and bark are the least used plant parts (Figure 2). The information on scientific name, local name of the plant, plant part used, and mode of preparation has been provided in alphabetical order in Table 1. The plant uses can be divided into four main categories, medicinal use (36 species), human food and food aromatizer (22 species), agricultural and veterinary use including plants as fodder (17 species), and domestic and handicrafts uses (16 species) (Figure 3). The plants are used medicinally for curing fever, cough, arthritis, joint pain, abdominal parasites, jaundice, snake bite, and a number of other diseases. Aerial parts (82.09%) are the most frequently used than the underground parts (17.91%). Despite the ban from the government, roots of *Aconitum violaceum, Angelica glauca, Berberis lycium, Inula racemosa, Picrorhiza kurroa, Podophyllum hexandrum, Saussurea costus, Fritillaria cirrhosa* and *Valeriana jatamansi* are exploited heavily and sold to the middlemen or local contractors to fulfill the other household needs. Formulations of these plants are prescribed in paste form, powder form, juice form, decoction form, bandages, and smoke form. Paste form is the most common type of formulation given while smoke is least used (Figure 4). Herbs are most frequently used followed by shrubs, trees, and climbers (Figure 5). *Angelica glauca*, *Artemisia maritima*, *Heracleum candicans*, *Origanum vulgare*, *Podophyllum hexandrum*, *Rheum australe*, *Thymus linearis*, and *Taraxacum officinale* are used for treating more than one ailment. Eight plant species are used along with others or with more than one ingredient. Leaves of *Stellaria media* and *Malva neglecta* are cooked as mixed vegetable and eaten two or three times to cure constipation. Aerial parts of *Thymus linearis* in combination with *Origanum vulgare *are crushed with water and juice is extracted and given 4-5 teaspoons orally three times a day for high fever in children. *Rubia cordifolia* with *Cynodon dactylon *(*Doob* grass) is used against snake bite while a decoction of *Viola canescens *with * Cinnamon*, *Fennel*, and *Clove* is recommended for cough, asthma, and other respiratory tract problems. Flowers of *Morina coulteriana *and *M. longifolia *are mixed with *guggal* (roots of *Jurinea macrocephala*) are used as incense for ritual performances and for pleasant aroma during meditation and prayer. By comparing the earlier reported ethnobotanical/ethnopharmacological uses/biological activities/chemical constituents (Table 1), we found that there are 35 plants with new medicinal and other important ethnobotanical use from the study area. Plants with additional new uses are *Aconitum violaceum, Angelica glauca, Artemisia maritima, Berberis lycium, Bergenia ligulata, Cicer microphyllum, Clematis grata, Crataegus songarica, C. oxycantha, Cuscuta reflexa, Datisca cannabina, Datura stramonium, Hedera nepalensis, Jasminum officinale, Lactuca dissecta, Lonicera quinquelocularis, Malva neglecta, Mentha longifolia, Morina longifolia, Onosma hispida, Origanum vulgare, Oxyria digyna, Polygonum alpinum, Prunus cornuta, Ranunculus laetus, Rubia cordifolia, Rumex acetosa, R. nepalensis, Sarcococca saligna, Stellaria media, Thymus linearis, Ulmus wallichiana, Valeriana jatamansi, Viburnum grandiflorum, Viola canescens, *and* Viscum album* reported here for the first time from the study area (new medical remedies or ethnobotanical uses were given with asterisk mark in Table 1). Survey from the Pangi Valley reveals that paste was prepared by grinding the fresh or dried plant parts with oil, churning curd water or cow urine. The powder was prepared by the grinding of shade dried plant parts. The decoction was obtained by boiling the plant parts in water until the volume of the water is reduced to the minimum or required amount. The plants used against snakebite are applied externally. Some plants like *Astragalus rhizanthus, Cicer microphyllum*, *Desmodium elegans, Hedera nepalensis, Impatiens sulcata, Lonicera quinquelocularis,* and *Morus serrata* were documented as fodder plants in this study.

## 4. Discussion and Conclusions

The widespread use of herbal materials for the maintenance of health and treatment of diseases can be traced back to prehistoric times throughout many cultures and regions. The history of herbal medicine in India is very old. The oldest use of plants has been documented in ancient Hindu scriptures like *Rigveda *(4500–1600 BC), *Charaka Samhita *(1000–800 BC), *Sushruta Samhita *(800–700 BC), and others. In India, the art of herbal healing has very deep roots in tribal culture and folklore. Even today, most of the tribal communities are dependent upon local traditional healing systems for their primary health care. Tribes of Pangi Valley depend highly on the wild plants for their livelihood security and medicines for various ailments. The ethnobotanical information discussed here is the first ever comprehensive ethnobotanical information gathered from the “*Pangwal*” tribe. The herbal medicines are considered to be of great importance among different rural or indigenous communities in many developing countries [56]. During the last few years, the use of herbal supplements increased from 2.5% to 12%. Today approximately 80% of the world's population uses traditional medicine for healthcare and therapeutic purposes [57]. The Himalayas, one of the world's biodiversity hot spots, have an approximately 10,000 species of plants, of which about 3,160 belonging to 71 genera are endemic. About 1,195 species of flowering plants are endemic to the Western Himalayas [58, 59]. Cultural diversity in such remote mountain regions is closely linked to biodiversity, as there is a symbiotic relationship between habitats and cultures and between ecosystems and cultural identity; indeed, religious rules and rituals often strengthen this relationship and are characterized by a conservation ethic [60]. Present study also reveals that there is a strong relationship between tribes of Pangi Valley and plants of their surroundings. From the time immemorial, these people were highly dependent upon plant resources of their surroundings to fulfil their day-to-day requirements. As these people are very close to nature due to their inhabitation in isolated and remote tribal area, they have been able to gain a very vast and authentic experience of plant resources of their surroundings, which further need detailed investigation of ethnopharmacological studies from this tribal area. Screening and comparing the literature regarding ethnobotanical studies from other parts of Himachal Pradesh [10, 14, 22, 27, 34, 36, 45, 47, 61, 62] and outside of Himachal Pradesh [19–21, 41, 44, 49] show a high number of species with unreported uses or new use with different part used and also new mode of use from the study area (Table 1). Pangi Valley is the semi-arid transition zone between the Northwest Himalaya and trans-Himalaya and thus has elements of both regions making the assemblage among the most diverse for any other region in the Northwest Himalaya. Because of its unique geographical situations, it harbours distinct ethnic and endemic biological diversity. This is one region in the country, where people still depend largely on plants for traditional healing system. A large number of plants/plant extracts/decoctions or pastes are equally used by tribes and folklore traditions in India for treatment of cuts, wounds, and burns [63–67]. Some of the plants such as *Onosma hispida* and *Ranunculus laetus* were reported for cuts and wounds from the research area are new to use. Smoke of flowers of *Morina coulteriana *and *M. longifolia *mixed with *guggal* (roots of *Jurinea macrocephala*) is used as incense during meditation and prayer. Use of pleasant aroma of smoke during meditation is also performed in Chinese culture [68]. Livestock is also considered one of the main sources of livelihood and important part of livelihood security, which rely mostly on fodder extracted from forests, grasslands, agriculture, and agroforestry in this interior remote tribal area. Some plants species such as* Cicer microphyllum*, *Desmodium elegans, Hedera nepalensis, Impatiens sulcata, Lonicera quinquelocularis, Morus serrata*, *Origanum vulgare, Rumex acetosa, Silene vulgaris, Smilax aspera,* and *Ulmus wallichiana* have also been documented as fodder plants in this study. Preparation of paste for the treatment of ailments is a common practice among the other tribal communities in India [69, 70]. Plant parts are used commonly for snake bite and such plants used against snake bite are also needed to be explored for more detailed studies [71, 72]. Present study explores information for the first time from the Pangi Valley on traditional therapeutic for joint pains, abdominal disorders, snake bites, skin disorders, cuts and wound, burns, high fever, cough, and many other diseases. This study contributes significant ethnobotanical information from the remote high altitude and difficult region of the world, which remained cut off from rest of the world for 6-7 months. Further investigations of those plants which are not explored earlier may lead to the exploration of several novel bioactive molecules and many new drugs to various diseases from such geographically isolated and unexplored area. The unsustainable harvesting of medicinal plants from the wild may cause a serious decline in plant population. It is thus recommended that cultivation techniques be designed, especially the important medicinal plant species that are used widely, to fulfil the need of the growing international herbal market and strategies to conserve the threatened biodiversity.

## Figures and Tables

**Table 1:** Ethnomedicinal and Ethnobotanical uses of plant species in Pangi Valley and its adjoining areas of district Chamba of Himachal Pradesh.

Botanical name, habit, and plant accession No.	Family	Local name	Part(s) used	Mode of preparation and uses recorded from Pangi valley	Earlier reported ethnobotanical/ethnopharmacological uses/biological activities/chemical constituents
*Angelica glauca* Edgew. (Herb), 58760	Apiaceae	Chura	Roots	Dried roots in powdered form are used for joint pains and in fever, Used to cure cough, gastrointestinal complaints, stomachache and rheumatism Roots are burnt to smoke and fumigations are used to keep snakes away from inhabitation. Powdered roots used as spice in various recepies to provide pleasant aroma and flavour to food*	Dysentery, gastric, stomach disorder, vomiting [11–14], Essential oils (*β*-phellandrene, *α*-cadinol), lactone, coumarin (I), isoimperatorin, prangolarin, furocoumarins [15], Roots are burnt to remove the snakes when they enter the house [16]

*Aconitum heterophyllum *Wall. ex Royle (Herb), 51397	Ranunculaceae	Atis	Roots	Root powder is used for fever and abdominal pain	Alkaloids, atisine, hetisine, heteratisine, atisenol, heterophyllisine [17], hetidine, atidine, hetisinone, benzothteratising, F-dihydroatisine [14, 15]

*Aconitum violaceum* Jacq. ex Stapf (Herb), 58295	Ranunculaceae	—	Roots	Dried roots in powdered form are used for joint pains*	Antipyretic, abdominal pain, antidote, anti-inflammatory [18]

*Artemisia maritima *L. (Herb), 58440	Asteraceae	Saici	Aerial parts	Decoction is prepared after boiling the aerial parts in water. Tonic used to remove abdominal parasites of children. Considered antiseptic blood purifier and vermifuge*	Gastric complaints [13, 19], abdominal pains [20], indigestion [12, 21], Anthelmintic [17]

*Artemisia parviflora *L. (Herb), 51734	Asteraceae	Shambar booti	Aerial parts	Decoction is used against stomachache also vermifuge; Paste is used for cuts and wounds	Asthma, epilepsy, nervous disorders, peptic ulcers, skin diseases, sores, insect repellent and stomachache. [13], The leaf paste is applied on cuts and wounds to check bleeding [22], Leaves contain essential oil up to 0.35%. Infusion of leaves given to asthma, nervous and spasmodic affections. Roots used as tonic and antiseptic [15]

*Astragalus himalayanus* Klotz. (Herb), 58789	Papilionaceae	Kayabachtp	Flowers Seeds	Powdered seeds and flowers given in strangury	Inhabitant of Lahaul-spiti also use powdered seeds and flowers given in strangury [14]

*Astragalus rhizanthus *Royle ex Benth. (Herb), 51203	Papilionaceae	Zomoshing	Roots	Roots used as fodder	Fodder [14]

*Berberis lyceum* Royle (Shrub) 58763	Berberidaceae	Kasmal	Roots and stem	Roots juice is used to cure eye infection Stems are used to brush the teeth to kill harmful bacteria*	To cure eye infection [23]

*Berginia ligulata* (Wall.) Engl. (Herb), 58784	Saxifragaceae	Shaprotri	Leaves	Leaves are ground and fumes are inhaled to recover from heavy sneezing. Leaves are used as “*Pattar*”, a kind of eco-friendly disposable plate, used during marriage and other ceremonies*	CaC(2)O(4) crystal inhibition, diuretic, hypermagneseuric and antioxidant effects and this study rationalizes. Its medicinal use in urolithiasis [24]

*Chenopodium album* L. (Herb), 58783	Chenopodiaceae	Baathu	Leaves and seeds	Used for both green and grain	Whole plant is used for ulcers, swellings and seminal weakness [25], indigestion [26] used for both for grain and green [27]

*Chenopodium foliosum* Wall. (Herb) 58779	Chenopodiaceae	—	Fruits	Red juicy fruits are eaten as it is	Indigestion [26], ripe fruits are mixed with grains for consumption [28], red juicy inflorescence is eaten [27]

*Cicer microphyllum *Benth. (Herb), 58785	Papilionaceae	Chiri	Leaves and stems	Used to cure mouth infection like mouth ulcer. Fodder is suited best for cow to increase milk yield*	Whole plant is used for increasing milk production and as general tonic for cows [25], sore mouth in cattle, tongue infection, jaundice [26]; immature are eaten, potential breeding material for cultivated* Cicer* [27]

*Clematis grata* Wall. (Herb), 58458	Ranunculaceae	Bharani	Leaves	Leaves are used for the eruptions of the pimples and boils. Leaves are crushed either with water or urine of cow and a paste is made and then the paste is applied on the infected part to cure the infected part*	Shoots used for ring worm, baldness, and as antimycotic [29]

*Crataegus songarica* K. Koch (Tree), 58778	Rosaceae	Pingyath	Fruits and wood	Ripe fruits are eaten by school children and road side laborours. Wood is used to make plough for field; fruits are sold to contractors at 18–20 Rs/kg	Fruits are edible and considered as cardio tonic. Wood is heavy, hard, and tough and is used for making tool hands, mallets, and other small items. Also used as fuel wood. Leaves are used for fodder [30]

*Cuscuta reflexa *Roxb. (Climber), 58484	Cuscutaceae	Amarbel	Whole plant	The plant is used in jaundice. Ladies used whole plant as hair tonic by macerating the plant in *Brassica *oil*	Whole plant extract is considered as antiviral [31] and analgesic [32], methanol extract of stem possesses antibacterial activity [32]

*Datisca cannabina* L. (Shrub), 58282	Datiscaceae	Pahari neem	Leaves	The leaves are used to protect clothes from worms*	Fever and gastric [26]

*Datura stramonium *L. (Herb), 58782	Solanaceae	Datura	Seeds	4-5 seeds are ground to powered form and added 10–15 liter of alcohol to increase the effect and properties*	Softening of the boils and earache [29]

*Desmodium elegans* DC. (Shrub), 58749	Papilionaceae	Kathi	leaves	Leaves are used as fodder	Carminative, tonic, diuretic, chronic fever, cough, vomiting, asthma, and in snakebite [29]

*Elaeagnus conferta *Roxb. (Tree), 58777	Elaeagnaceae	Gaihein	Fruits	Fruits are eaten	Faster clearance of blood alcohol after the alcohol ingestion [33]

*Epilobium aungustifolium *Lam. (Herb), 51634	Onagraceae	Dharshak	Roots	Pulverised roots are used as detergent	Pulverised roots are used as detergent [14]

*Gentiana moorcroftiana *Wall. ex G. Don (Herb), 58491	Gentianaceae	—	Leaves	Effective for liver problem	Jaundice [34]

*Hedera nepalensis* C. Koch (Climber), 58776	Araliaceae	Kurrai	Leaves with stems	Leaves are considered as tonic for cattle so used as fodder*	The dried branches and leaves are ground and the powder is used early in the morning with water against diabetes [35]

*Heracleum lanatum* Michx. (= *H. candicans *Wall. ex DC.) (Herb), 58489	Apiaceae	Dundu	Roots	Grounded root paste is used in snake bite*. It is used to treat fever and abdominal cramps caused by the intestinal worms	Plant is a good fodder for goats which increases milk production and medicinally it is used for nerve disorders and sexual problems [25]

*Impatiens sulcata *L. (Herb), 58718	Balsaminaceae	Halva	Seed and leaves	Seeds are eaten by school children and road side laborers. Plant is sun dried and stored as a fodder with other grasses for winter season for consumption to domestic cattle*	Urticaria, eczema, pimples, and abortifacient [26]

*Inula racemosa* Hook. f. (Herb), 58787	Asteraceae	—	Roots	It is used to treat asthma, treat stomach disease, rheumatism, liver complaint	Paste of roots is used to cure boils [36], growing wild earlier, now a cultivated crop of the region [27]

*Jasminum officinale* L. (Shrub), 58759	Oleaceae	Swain	Leaves and Stems	Leaves and stems are used in the marriage ceremonies as aesthetic value and others are used for religious purposes*	Leaves and flowers are used for cough, fever, and as blood purifier [29]

*Lactuca dissecta *D. Don (Herb), 58602	Asteraceae	Dudhil	Leaves and stems	Paste is used to cures infections of female external genital organs*	Allelopathic potential [37]

*Lonicera quinquelocularis* Hardw. (Shrub), 58426	Caprifoliaceae	Bakhur	Stems, leaves and fruits	Fruit juice is applied to cure cracks of foot and hands and is also used as fuel. Leaves are used as fodder*	A new iridoid glycoside 6′-O-beta-apiofuranosylsweroside was isolated from the ethanolic extract of the roots along with the known compounds loganin and sweroside [38]; fresh leaves are crushed and the extract is poured in eyes to cure the cataract and to improve vision. Fresh leaves are used as fodder for goats [35]

*Malva neglecta* Wall. (Herb), 58420	Malvaceae	Sonchal	Leaves	Leaves of *Stellaria media* and *Malva neglecta* are cooked as mixed vegetable and eaten two or three times to cure constipation*	Malaria, bladder, kidney disorder, laxative [26], and antiobesity [39]

*Mentha longifolia *(L.) Huds. (Herb), 58771	Lamiaceae	Marhendri	Leaves with stems	A paste is also made from the leaves and applied to burst the boils for pus removal. Leaves are placed inside the container containing seeds to kill and prevent the attack of insects on the stored seeds; leaves with stems are also placed inside the catteries to protect them from ticks, mites, and rat flea*	Stomach problems, carminative, liver problems, vomiting and indigestion [25], *cis*-piperitone epoxide, piperitenone oxide, carvone, menthone, thymol,pulegone *β-*thujone, (*E*) caryophyllene, myrcene, carvacrol, borneol, and *p-*cymene [40]. Medicinal use in diarrhoea and gut spasm, calcium channel blocking activity [41], insecticidal properties [42]

*Morina coulteriana *Royle (Herb), 58772	Morinaceae	Tinglaa	Flowers	Flowers are mixed with guggal (Roots of *Jurinea macrocephala*) for incense because of pleasant aroma	Eye complaints [26]

*Morina longifolia *Wall. (Herb), 58773	Morinaceae	Tinglaa	Flowers	Flowers are mixed with guggal (Roots of *Jurinea macrocephala* and flowers of *Morina coulteriana*) for incense due to pleasant aroma*	The root powder is applied as poultice in boils for sucking the puss out of it and facilitating healing of the wounds [22]. Boils [13], used as incense in the preparation of dhoop and agarbattis and so forth yield an essential oil [15]

*Morus serrata* Roxb. (Tree), 58751	Moraceae	Kruum	Fruits and Wood	Fruits are eaten and leaves are used as fodder; wood is used to make furniture	The fruits are edible and are used as digestive stimulant and to relieve constipation and other digestive problems. The leaves are used for fodder. Wood is used for furniture and fuel [35]

*Onosma hispida *Wall. ex G. Don (Herb), 58453	Boraginaceae	Kom	Roots and leaves	Used for cuts, swells, wound, and ulcer. Lama (Priest or Bhot people) use the dye for religious ceremonies*	Root extract is used for pneumonia and typhoid fever and also used for dyeing hairs [25], stimulant, blood purifier, cuts, swelling, ulcers [26]

*Origanum vulgare* L. (Herb), 58774	Lamiaceae	Marua	Leaves, and stems	Aerial part of *Origanum vulgare *in combination with *Thymus linearis* is crushed with water and juice is made, given 4-5 teaspoon orally three times a day during high fever in children; this is very effective medication. Also used as a coolant. Also used as fodder*	Paste of leaves and terminal shoots along with 2-3 fruits of black pepper (*Piper nigrum*) is applied to boils, ulcers, wounds, cuts, and weeping eczema. Paste of leaves is reported to be useful in healing the wounds caused by fire burns. The root pieces of plant are bound in a cloth piece and tied to the necks of infants as a protective measures against conjunctivitis [22], cold, fever, hysteria, menstrual complaints, and tonic [13]; leaves and tops cut prior to blooming are used as a flavouring agent; origanum oil is carminative stomachache, diuretic, diaphoretic, and emmenagogue and is used as a stimulant and tonic in diarrhoea. Given in whooping cough and bronchitis because of its spasmolytic action, also employed in cosmetics and soaps [15]

*Oxyria digyna* (L.) Hill (Herb), 58775	Polygonaceae	Suchali	Leaves	Leaves and inflorescence are edible	Whole plant is used for appetite, fever, laxative [26], and leaves, and inflorescence are edible [27]

*Phytolacca acinosa* Roxb. (Herb), 58756	Phytolaccaceae	Ranshag, Ashlu	Leaves	Young tender leaves are used for the preparation of vegetable	Fresh leaves are boiled and consumed to relieve bodyache and diarrhoea [43]

*Picrorhiza kurroa* Royle ex Benth (Herb), 58764	Scrophulariaceae	Kour	Roots	5–10 gm of dried powder is taken with water, two times a day, to relieve from joint pains	To cure anaemia, asthma, diarrhoea, jaundice, promotes secretion of bile and used in stomach diseases [13]; roots are used in abdominal pains and as a purgative too. One to two leaves are crushed and drops of the juice are poured in the nose to stop bleeding [22], constitute the drug picrorhiza, and are used as a substitute of Indian Gentian (*Gentiana kurroo*) containing picrorhizin, kutkin, and other compounds [15]

*Podophylum hexandrum* Royle (Herb), 58752	Berberidaceae	Bankakri	Roots Fruit	Roots are dried and used in powered form for joint pains, arthritis, and asthma, Fruits are edible	The root powder is administered internally for gastric ulcers. It is applied as a paste on cuts and wounds for regeneration of the tissues. Decoction of roots is used to cure liver problems [27] and hepatic diseases [13] Fruits are edible [15], diarrhoea [13, 44–46], blood diarrhoea [34, 47], chronic constipation [14], anticancer [17] and they constitute a compound called, podophyllin, which is commonly used as a purgative; podophyllotoxin is the active principle. Podophyllin is an effective vermifuge. Recently it has acquired importance because of its possible use in controlling some forms of cancer.

*Polygonum alpinum *Allioni.(Herb), 58786	Polygonaceae	Chohr	Stem and leaves	Tender stems are eaten raw to cure the cracks of lips and gums. Leaves are used as fodder*	Cough, dysentery, haemostasis, tonic, abortion, wounds, and heart burn [26]

*Potentilla nubicola *Lindl. ex Lacaita *(Fragaria nubicola *Hook.) (Herb), 58753	Rosaceae	Dhul-akhre	Fruits	Fruits are collected and eaten raw	Fruits and leaves are used as carminative, for stomach ulcers, and as antiseptic [29]

*Prunus cornuta* (Wall. ex Royle) Steud. (Tree), 58762	Rosaceae	Jammu	Fruits and Stem	Fruits are eaten. Stems are used for making many agricultural tools and also used as fuel. It is reported that the leaves are avoided to cattle fodder as the leaves are considered very poisonous and kill the cattle*	Rheumatism and wounds [26]

*Ranunculus arvense *L. (Herb), 58614	Ranunculaceae	Gudi	Leaves	Paste of leaves cures cuts or wounds by drying pus	Counter-irritant, anthelmintic, cooling, emollient and for wounds [26]

*Ranunculus laetus *Wall. ex Royle (Herb), 58290	Ranunculaceae	Jaldaru	Leaves	Paste is applied on cuts and wounds*	Antimicrobial activities [17]

*Rheum australe *D. Don (Herb), 58765	Polygonaceae	Chukari	Roots and leaves	Roots are sun dried and ground to powered form, then by adding water a thick paste is made and paste is then applied on the cuts and wounds for healing. Leaves are dried and ground with wheat flour for use during winters	Used as astringent, laxative [15], asthma, cough, fever, piles, skin diseases, ulcers, and wounds [13]; the paste of the root mixed in water is applied externally in muscular injury, cuts, wounds, and mumps and to forehead in headache. The watery extract is given orally in stomach pains, constipation dysentery, swelling of the throat and tonsillitis. Lotion is dropped in ears in earache [22]; leaves are dried and ground with wheat flour for use during winters [27]

*Rubia cordifolia* L. (Herb), 58780	Rubiaceae	Mishtu	Leaves and stems	A paste of *Rubia cordifolia* and *Cynodon dactylon *(Doob grass) is applied around the snakebite*	Root decoction with water is given to cure urinary infection; paste is used as an ointment to skin diseases. Root is also used to make dyes [43]; roots are used for blood purification, liver problems, swellings, nervous disorders, gouts, rheumatism, uterine tumors, bleeding control, leucorrhoea, wounds, cough, bone fractures, and general debility [25]

*Rubus ellipticus* Sm. (Shrub), 58754	Rosaceae	Aakhre	Fruits	Fruits are eaten by the local people	Fruit is edible and is having cooling effect. Spiny branches are used as fence around fields. Leaves are browsed by goats [35]. Young shoot is chewed raw to relieve sudden stomach pain. Root decoction is given to the children to get rid of stomach warm. Root paste is applied on forehead during severe headache; fruit is edible [43]

*Rubus niveus* Thunb. (Shrub), 58755	Rosaceae	Lal aakhre	Fruits	Fruits are eaten	Fresh root tips are used for curing excessive bleeding during menstrual cycle [23]

*Rumex acetosa *L. (Herb), 58634	Polygonaceae	Podoi	Leaves	Leaves are collected and used as vegetable, Eaten as a leaf vegetable. Used as a good fodder for cattle*	Jaundice, vomiting, liver problems [25], cuts, wounds, and nettle sting [26]

*Rumex nepalensis* Spreng. (Herb), 58781	Polygonaceae	Ubbal	Leaves	Leaves are crushed and solution is made and used as pesticide to kill pests. Also leaves are crushed and paste is made with milk, churned curd, or with the urine of cow and applied on the area around the snake bite on the body*	Juice is prepared by smashing leaves and young shoots are applied to heal wounds. Root is crushed and the juice applied on the scalp prevents hair loss [43]; roots are boiled in water and applied externally for swellings and joints pain [25]; leaves are crushed and applied on wounds as an ant allergic [23]

*Sarcococca saligna *(D. Don) Muell.-Arg. (Shrub), 58767	Buxaceae	Diyund	Leaves and Stems	Leaves are ground and paste is applied on the burns for quick relief. Paste acts as coolant. Stem is used as fuel and leaves in the ceiling of roof of houses as a waterproof medium*	Aqueous extract is used as antipyretic and calmative [48]

*Saussurea costus *(Falc.) Lipsch. (Herb), 58439	Asteraceae	Kuth	Roots	Plant roots are used in the treatment of cold and also for joint pain. Dried roots are ground to powdered form and taken orally	Joint pains [45], rheumatism [13, 14, 41, 49], spasmogenic, hypotensive, bronchodilatory, duretic [15], and CNS depressant [17]. Roots are internally used for asthma, cough, paralysis, brain problems, nervous problems, rheumatism, gouts, throat problems, and influenza and as a sex stimulant [25]; root paste is applied externally to cure joint pains [23]

*Scorzonera virgata *DC. (Herb), 58433	Asteraceae	Thunbu	Leaves	Leaves are used to cure constipation	Leaves are used to cure constipation [14]

*Silene vulgaris* (Moench) Garcke (Herb), 57383	Caryophyllaceae	Ghantolu	Leaves	Tender leaves are cooked as vegetable. Plants are also used as good fodder source	Leaves and twigs and used as pot herb [14] and for bronchitis and asthma [26]

*Smilax aspera* L. (Shrub), 58758	Smilacaceae	Dadrund Thuthur	Fruits and leaves	Fruits are eaten while leaves are used as fodder	Diuretic, diaphoretic, and arthritis [26]

*Stellaria media* (L.) Vill. (Herb), 58415	Caryophyllaceae	Kokuwa	Leaves	Leaves of *Stellaria media* and *Malva neglecta* are cooked as mixed vegetable and eaten two or three times to cure constipation*	Burns, boils, bone fracture, and wounds [26]; leaf paste of the plant is also applied on wounds caused by burning [23]

*Taraxacum officinalis *Wigg. (Herb), 58287	Asteraceae	Dudhi	Leaves, Roots	Leaves are used as bandage on cuts. Root powder is used against headache and fever. It is also used to cure jaundice	Liver complaints [13], jaundice, liver problems [46], rheumatic pains [45]; BA-hypoglycemic, antitumor [17], germacranolide acids, glucans, mannan, proteins, scopoletin, esculetin [15], diester of taraxanthin, lactupicrin, triterpenes [50], and fresh and dried rhizomes constitute the drug, The rhizomes roots and leaves are eaten as salad, used in soups, and cooked as vegetable. Leaves and open flowers are used in the manufacture of beer, wines, and other diet drinks [15]. Blood purifier, dislocation of joints, dysentery, gastric, ulcers, kidney diseases, and liver complaints [13], taraxacin, taraxacerin, phytosterols, taraxasterol and homo-taraxasterol [14]; whole plant is crushed into a mesh and given internally in snakebite. The paste is also applied externally on the wound. Leaves are effectively used for fomentation in swollen parts, boils, and sprains [22]

*Thymus linearis* Benth. (Herb), 58770	Lamiaceae	Sunouni	Aerial parts	Aerial part of *Origanum vulgare *in combination with *Thymus linearis* is crushed with water and juice is made, given 4-5 teaspoon orally three times a day during high fever in children; this is very effective medication. Juice extracted by crushing is taken orally*	Stomachache [13], gastric trouble [45], stomach disorder [46, 51], spasmolytic, CNS active [17], terpenes, thymol, monoterpenoid geraniol, *α*-pinene, *β*-pinene, camphene, car-3-ene, limonene, *γ*-ter-penene, terpinolene, citronellal, trans-*β*-terpineol, carvacrol, bornyl acetate, linalool [15], and methyl carvacrol [50]

*Trigonella emodi *Benth. (Herb), 51158	Papilionaceae	Kuchona	Young leaves and stems	Tender shoots are used as vegetable	Shoots are used as vegetable [14]

*Ulmus wallichiana* Planch. (Tree), 58757	Ulmaceae	Mandhu	Leaves, bark and stems	Used for making the traditional footwear named as “Pule.” Stems are used for fuel; leaves used as fodder*	Fracture and dislocation of joints [26]

*Urtica dioica* L. (Herb), 58429	Urticaceae	Ain	Leaves	Leaves are cooked and eaten as vegetable	Neutral and acidic carbohydrate protein polymer, glycoprotein [15]. Root and seed decoction is taken to treat diarrhoea and cough. Curry, prepared using shoot tips, is given to female during child delivery as their slipperiness is believed to help delivering child. Rheumatism [13], gout [10], antidiabetic, anticancerous, antianaemic, muscle stimulant [17], vitamin and carotenes, betaine, choline, and amino acids [17]

*Valeriana jatamansi *DC. (Herb), 58769	Valerianaceae	Shamak, Mushakwala	Roots	Skin disorder is cured. Added in incense for better aroma*	*α*-bulnesene, *α*-guaiene, guaiol, seychellene, viridiflorol, and *β*-gurjunene [52]

*Verbascum thapsus *L. (Herb), 58300	Scrophulariaceae	Jangli Tamaku	Flower and leaves	Paste of flower and leaves is applied on boils	Leaves and fruits are used in diarrhoea and pulmonary disease of cattle. Leaves are also used as demulcent, in pectoral complaints and as local application in piles, sunburns and inflammation of mucus membrane. Dried leaves are smoked and relieve irritation. Decoction of the leaves is used as a heart stimulant. Roots show febrifuge properties [15], asthma, cough, and fish poison [13]; crushed leaves are given in constipation and allied stomach pains [22]

*Viburnum cotinifolium *D. Don (Shrub), 58600	Caprifoliaceae	Ka	Fruits	Ripe fruits are edible raw	Fruit is considered to be laxative and blood purifier. Leaves extract is applied in menorrhagia [48]

*Viburnum grandiflorum *Buch-Ham. ex D. Don (Shrub), 58768	Caprifoliaceae	Tilhanj	Fruits and stem	Ripe fruits are eaten raw and stem is used as fuel*	Seed juice is given to treat whooping cough and typhoid [53]

*Viola canescens *Wall. ex Roxb (Herb), 58766	Violaceae	Ratmundi/Vanksha	Flowers	Decoction of flowers with cinnamon, fennel, and clove is recommended for cough, asthma, and other respiratory tract problems*	Antimalarial [52] and antiplasmodial activity [54]; leaves paste is mixed with brown sugar to be used against cough, cold, and other respiratory problems [35]

*Viscum album *L. (Climber), 58750	Loranthaceae	Ranau	Bark	A paste of bark is used on the fresh burns for the healing. It is highly useful in healing the deep wounds caused by fire burns*	Decoction made from whole plant is used for enlarged spleen [55]

*New medical remedies or ethnobotanical uses.
